# Neutrophil Extracellular Traps and Their Implications in Cardiovascular and Inflammatory Disease

**DOI:** 10.3390/ijms22020559

**Published:** 2021-01-08

**Authors:** Johannes Klopf, Christine Brostjan, Wolf Eilenberg, Christoph Neumayer

**Affiliations:** Division of Vascular Surgery and Surgical Research Laboratories, Department of Surgery, Medical University of Vienna, General Hospital of Vienna, 1090 Vienna, Austria; johannes.klopf@meduniwien.ac.at (J.K.); christine.brostjan@meduniwien.ac.at (C.B.); wolf.eilenberg@meduniwien.ac.at (W.E.)

**Keywords:** neutrophil extracellular traps (NETs), neutrophils, cardiovascular diseases, inflammation, autoimmunity, atherosclerosis, abdominal aortic aneurysm, diabetes mellitus, COVID-19, malignant neoplasia

## Abstract

Neutrophils are primary effector cells of innate immunity and fight infection by phagocytosis and degranulation. Activated neutrophils also release neutrophil extracellular traps (NETs) in response to a variety of stimuli. These NETs are net-like complexes composed of cell-free DNA, histones and neutrophil granule proteins. Besides the evolutionarily conserved mechanism to capture and eliminate pathogens, NETs are also associated with pathophysiological processes of various diseases. Here, we elucidate the mechanisms of NET formation and their different implications in disease. We focused on autoinflammatory and cardiovascular disorders as the leading cause of death. Neutrophil extracellular traps are not only present in various cardiovascular diseases but play an essential role in atherosclerotic plaque formation, arterial and venous thrombosis, as well as in the development and progression of abdominal aortic aneurysms. Furthermore, NETosis can be considered as a source of autoantigens and maintains an inflammatory milieu promoting autoimmune diseases. Indeed, there is further need for research into the balance between NET induction, inhibition, and degradation in order to pharmacologically target NETs and their compounds without impairing the patient’s immune defense. This review may be of interest to both basic scientists and clinicians to stimulate translational research and innovative clinical approaches.

## 1. Introduction

Neutrophils represent 50–70% of circulating leukocytes in healthy adults and are thus the most common and central cells of the non-specific, innate immune response [1,2]. At the site of inflammation, neutrophils can act as signal mediators and, if activated, perform various antimicrobial functions and tasks including phagocytosis, cytokine secretion, and degranulation [3,4,5]. Recently, the formation of so-called neutrophil extracellular traps (NETs) has been described as a new defense mechanism [6,7]. NETs are net-like complexes consisting of chromatin DNA, histones, and neutrophil granule proteins which are released to the extracellular space. They most likely represent an evolutionarily conserved element of the unspecific immune response and bind pathogens to prevent their spread and ensure their elimination through increased local concentration of antimicrobial and toxic factors. The process of activation and release of NETs is generally referred to as NETosis [8,9]. However, besides the desired antimicrobial function, NETs may also contribute to the acute or chronic pathogenesis of various diseases, especially vascular and immune-related diseases [10]. The following article provides an overview of the mechanisms of NETosis and the established roles of NETs in the course of various diseases, with a focus on the importance of NETs in cardiovascular and inflammatory disorders. This review may be of interest to both basic scientists and clinicians to stimulate translational research and innovative clinical approaches.

## 2. Cellular Defense Functions of Neutrophils

Neutrophils as the most abundant circulating leukocytes in the human immune system are typically recruited as the first effector cells to an inflammatory site, which is considered an essential step for the rapid clearance of infections [5]. In line, congenital neutropenia is associated with severe immunodeficiency in humans [11]. In healthy adults, the production of neutrophils reaches up to 2 × 10^11^ cells per day and is controlled by granulocyte colony stimulating factor. Granulopoiesis starts from the unipotent myeloblast stem cell in bone marrow and ultimately results in the polymorphonuclear, mature neutrophil cell with an average diameter of 7–10 µm, a segmented nucleus, and cytoplasm enriched with distinct granules and secretory vesicles [12]. During maturation, three types of consecutively formed neutrophil granules have been identified and classified based on the presence of characteristic granule proteins. These are primary (azurophilic, peroxidase-positive) granules, which contain myeloperoxidase (MPO), neutrophil elastase (NE), proteinase 3, cathepsin G, various defensins, and azurocidin, secondary (specific, peroxidase-negative) granules carrying effector molecules like lactoferrin, cysteine-rich secretory protein 3, cathelicidin LL-37, and lipocalin 2, as well as tertiary granules, which are filled with arginase 1 and gelatinases like matrix metalloproteinase 9 [13,14,15]. Human neutrophils additionally contain easily mobilizable secretory vesicles which are functionally distinguishable from azurophilic, specific, and tertiary granules. These secretory vesicles transport proteins to the cell surface which are essential for cell adhesion such as integrins but also alkaline phosphatase and proteases to facilitate transmigration and efficient immune defense [13,16].

Thus, neutrophils can eliminate pathogens by distinct means, either by phagocytosis using ROS-dependent mechanisms or antibacterial proteins such as cathepsins, defensins, lactoferrin, or lysozyme, which are released into the phagosomes (phagocytosis) or into the extracellular space (degranulation). In addition, activated neutrophils may abandon their cellular integrity and form so-called NETs to eliminate pathogens by immobilization and extracellular destruction, thereby preventing bacterial dissemination [14].

## 3. NET Morphology and Mechanisms of NET Formation

Neutrophil extracellular traps were first described in 1996 [7]. High-resolution scanning electron microscopy has shown that NETs consist of long fibers with diameters of 15 to 17 nm and globular domains ranging from 25 nm to larger aggregated complexes of up to 50 nm. Of note, NETs can occupy a space 10 to 15 times larger than the volume of the cells from which they originate [8]. In addition to the main components, nuclear DNA and histones, especially H3 and H4, these net-like structures are also composed of germicidal proteins from neutrophil granules such as MPO, NE, cathepsin G, and gelatinase. However, no cytoplasmic functional proteins such as actin, annexin I, and tubulin or integral transmembrane proteins such as CD63, have been found to be involved in NET complexes [6].

Various stimuli have been reported to induce NETosis. Neutrophil extracellular traps as part of the innate immune defense are released after infection with gram positive and negative bacteria, but also in particular by large pathogens and fungi [17]. The size of the microorganisms, their distinct virulence factors, and released inflammatory molecules are regulators of NET induction. Neutrophils usually remove small microorganisms by phagocytosis and fusion with their granules. Larger microorganisms which are not readily digested, lead to blocking of phagocytosis and commitment of cells to NET formation by promoting the cytosolic release and nuclear translocation of NE with subsequent actin cytoskeleton degradation as well as chromatin decondensation [9]. In addition to microorganisms and lipopolysaccharides, NET formation is triggered by other distinct stimuli including nitric oxide, urate crystals, autoantibodies, proinflammatory cytokines such as interleukin (IL) 1β, IL-6, IL-8, tumor necrosis factor-α (TNF-α), and the interaction of neutrophils with activated platelets or endothelial cells [18]. Also, the size of non-microbial, sterile particles influence NET formation, thus larger, cuspated urate crystals trigger NETosis more potently than small urate aggregates [19]. Examples for pathological, disease-specific triggers, which are manifold and variable, are summarized in Table 1.

For in-vitro studies, phorbol-12-myristate-13-acetate (PMA), a cell-permeable activator of protein kinase C, calcium ionophores such as A23187 or ionomycin, hydrogen peroxide, lipopolysaccharides, or the abovementioned cytokines are most often used as inducers of NETosis [17]. Efficiency and mechanism of NET induction varies with the applied stimulus as briefly outlined below [17].

**Figure 1:**
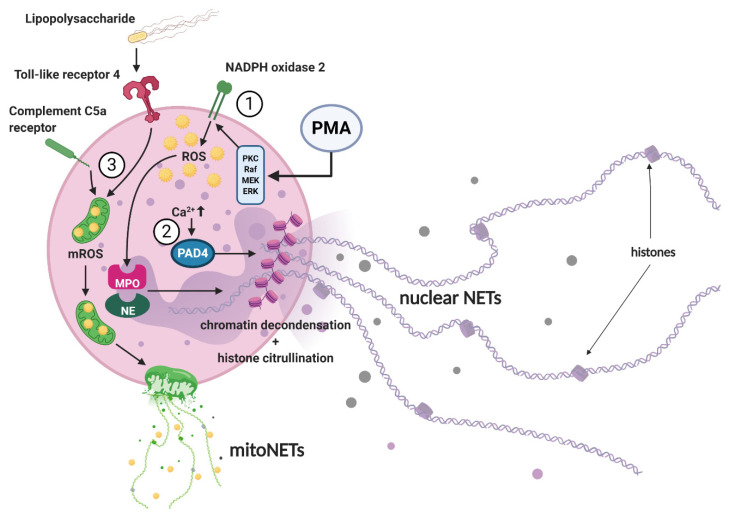
Mechanisms of NET formation.

Three main signaling pathways of NETosis were described by in-vitro experiments (Figure 1). After stimulation of neutrophils by PMA, a signaling cascade mediated by NADPH oxidase 2 (Nox2) is triggered and induces NET formation via the production of reactive oxygen species (ROS) [45,46]. ROS formation further promotes the translocation of MPO and NE, two key enzymes which are stored in azurophilic granules of naïve neutrophils, into the cell nucleus and causes the release of so-called nuclear NETs, which consist predominantly of nuclear DNA. MPO converts hydrogen peroxide to hypochlorous acid, activating NE, which in turn degrades the cytoskeleton and dismantles the nuclear membrane, allowing for NET expulsion. In addition, as an alternative pathway, histone citrullination and chromatin decondensation can be triggered independently of Nox2 by the activation of protein arginine deiminase 4 (PAD4) [45,47,48,49]. This pathway is, for example, initiated by calcium ionophores such as A23187 or ionomycin, which increase the intracellular calcium level and thus subsequently activate PAD4 [50]. Another form of NETosis has been described after priming neutrophils with granulocyte and macrophage colony-stimulating factor and subsequent short-term toll-like receptor 4 (receptor for lipopolysaccharide) or complement factor C5a receptor stimulation. The expelled NETs then contain oxidized mitochondrial rather than nuclear DNA [51]. These NETs are commonly referred to as mitoNETs. Remarkably, it was found that nitric oxide and PMA-induced NET formation leads to both, nuclear and mitochondrial NETosis [52]. The identified mechanism of NET formation has recently been reviewed in more detail by Castanheira and Kubes, by Rosazza et al. and by Van Avondt and Hartl [45,53,54].

Although different mechanisms of NET formation can be distinguished in-vitro, little is known about the engaged pathways and potential cross-stimulation in-vivo. This is currently one of the central unresolved research questions in the context of NET release. It seems likely that combinations of NET pathways are triggered in parallel. This is also supported by the fact that in patients with distinct diseases certain pathway-specific NET markers such as NE-DNA, MPO-DNA complexes, citrullinated histone H3 and H4, but also oxidized mitochondrial DNA are often detected concomitantly and are highly correlated. This phenomenon has been observed in severely ill cancer patients as well as in cardiovascular diseased patients with severe coronary atherosclerosis, abdominal aortic aneurysms, autoimmune small-vessel vasculitis, or systemic lupus erythematosus [26,31,55,56,57].

Different stimuli applied in-vitro have been shown to activate distinct intracellular pathways that lead to NET formation. ① The stimulation of neutrophils by PMA via the PKC and Raf-MEK-ERK signaling pathways leads to the activation of Nox2 resulting in cytosolic or mitochondrial ROS production (Nox2 dependent mechanism) which promotes the release of NE and MPO from neutrophil granules. After breakdown of the granule and nuclear membranes, MPO and NE translocate into the cell nucleus and induce chromatin decondensation, thus releasing nuclear NETs. ② Elevated intracellular calcium levels (e.g., initiated by calcium ionophores such as A23187 or ionomycin) activate the PAD4 enzyme which translocates into the nucleus and drives histone citrullination and chromatin decondensation (Nox2 independent mechanism) by promoting the weakening of the electrostatic binding between histones and DNA within nucleosomes. ③ The formation of so-called mitoNETs is mainly achieved by mitochondrial ROS production or after receptor stimulation of toll-like receptor 4 (e.g., by lipopolysaccharide) or complement factor C5a. Mitochondria translocate to the cell surface before they disintegrate and release oxidized mitochondrial DNA into the extracellular space. PMA: phorbol-12-myristate-13-acetate; PKC: protein kinase C; Raf: rapidly accelerated fibrosarcoma; MEK: mitogen-activated protein kinase kinase; extracellular signal-regulated kinase; ROS: reactive oxygen species; mROS: mitochondrial reactive oxygen species; PAD4: protein arginine deiminase 4; MPO: myeloperoxidase; NE: neutrophil elastase.

## 4. NET-Associated Diseases

In addition to the progress in knowledge about the specialized immunoprotective functions of NETs, recent research in animal models and in-vitro experiments is providing increasing evidence for the central pathophysiological role of NETs in disease. There is a remarkable spectrum of cardiovascular, inflammatory, autoimmune and metabolic diseases, infectious diseases, and certain septic conditions in which NETs seem to contribute to morbidity and mortality [34].

While multiple inducers of NETosis have been characterized in-vitro, various triggers of NET formation have also successfully been detected in-vivo. The diversity of NET inducers in distinct diseases has improved our understanding of their role in the different pathological conditions. Table 1 summarizes some of the known pathological and disease-specific triggers of NET formation.

### 4.1. NETs in Atherosclerosis

Atherosclerotic diseases are the dominating leading causes of mortality globally [58,59]. The development of atherosclerosis, starting with fatty streak formation and progressing to atheroma and plaque formation, relies on a lipid-driven chronic inflammatory process, involving vascular and immune cells [60]. It is known that hyperlipidemia can induce neutrophilia, which was shown to be positively correlated with atherosclerosis and atherosclerosis-related diseases in humans [61,62]. In line, the atherosclerotic plaque size in apolipoprotein E-deficient (ApoE^−^/^−^) mice was found to be closely correlated with the circulating neutrophil count [63]. Hyperlipidemia leads to damaged endothelial cells, promoting lipid deposition and subsequent plaque formation, which represents the onset of atherosclerosis. Since neutrophils were found to contribute to atherosclerosis by causing endothelial damage and propagating leukocyte recruitment into the lesion, also NETs have been investigated and found in-vivo in an atherosclerotic environment of the murine carotid bifurcation. Complementary to this finding, NETs were detected in human plaques obtained by endarterectomy [64]. Another study analyzing human carotid endarterectomy specimens showed that NETs were present predominately in superficial erosions near clusters of apoptotic endothelial cells as opposed to lipid-rich, vulnerable plaques [65]. In line, this finding was confirmed in atherosclerotic lesions of coronary arteries from hearts of cardiac transplant recipients [66]. In a murine model it was shown that sterile inflammation in atherosclerosis drives the production of cytokines, that trigger neutrophils to release extracellular traps [21]. Interleukin-1β plays a central role as a NET inducer in this context: Crystalized cholesterol is engulfed by monocyte-derived macrophages and activates their inflammatory response to release cytokines including interleukin-1β. Furthermore, the activation of Th_17_ cells amplifies immune cell recruitment and triggers NETosis in cholesterol-rich areas of atherosclerotic lesions. Since cholesterol crystals can also directly induce NETosis, it was shown that signaling steps of NETosis such as ROS burst and translocation of NE to the nucleus constitute the underlying intracellular mechanism, when human blood-derived neutrophils interact with cholesterol crystals. In line, NADPH oxidase inhibitor diphenylene iodonium or the inhibitor of NE and proteinase 3 successfully block ROS dependent NET formation and NE translocation to the nucleus driven by cholesterol crystals in-vitro. Thus, ApoE/NE/proteinase 3 deficient mice showed an approximately 3-fold reduction in atherosclerotic plaque size compared to ApoE knockout controls. Furthermore, degradation of NETs by DNase I treatment in ApoE^−^/^−^ mice resulted in a comparable 3-fold reduction in atherosclerotic lesion size, but in ApoE/NE/proteinase 3 deficient mice that lack NETs, the atherosclerotic plaque size was unaffected by DNase I treatment [21]. These findings indicate the importance of NETs in atherosclerotic disease which is supported by other studies showing that PAD4 inhibition via chloro-amidine not only decreases NETosis, recruitment of neutrophils and macrophages to arteries, but also reduces the atherosclerotic lesion size and delays the time to carotid artery thrombosis [67,68].

Since NETs are implicated in atherosclerosis and atherosclerosis-related diseases, their constituents can serve as putative biomarkers at a diagnostic or prognostic level to predict the severity of atherosclerosis and the risk of future cardiovascular events [56]. A prospective, observational, cross-sectional cohort of 282 individuals with severe coronary atherosclerosis showed significantly elevated levels of considered biomarkers of NETosis such as circulating cell-free, double-stranded DNA, nucleosome fragments, and MPO-DNA complexes when compared to healthy controls. Especially, the plasma levels of nucleosomes were found to be an independent surrogate marker of severe coronary stenosis. In addition, concentrations of MPO-DNA complexes correlated with the number of atherosclerotic coronary vessels and predicted the occurrence of major adverse cardiac events [56].

### 4.2. NETs Promote Vascular Disease

Cardiovascular diseases, in part also related to atherosclerosis such as arterial thrombosis, also venous thrombosis, among the world’s leading causes of morbidity and mortality, are associated with NETs and their interplay with the activation of intrinsic and extrinsic coagulation pathways [69,70]. Hypoxia-induced endothelial release of P-selectin and von Willebrand factor contributes to the recruitment and activation of neutrophils, whereby released NETs form a scaffold for binding of platelets, erythrocytes, fibrin, and coagulation factors, which was found to promote deep vein thrombosis [71,72,73]. Neutrophils also increase the production of thromboxane A2, which increases neutrophil interactions with the endothelium via the enhanced expression of intercellular adhesion molecule 1 [74] This process further stimulates NETosis via interactions with integrins, reactive oxygen species and the so-called high mobility group protein B1 [9,72,75]. On the one hand, NETs contribute to thrombosis or coagulopathy by activating coagulation factor XIIa and mobilizing Weibel–Palade bodies [75] On the other hand, NET-bound neutrophil elastase (NE) cleaves tissue factor pathway inhibitor, a factor that inhibits clotting, and enhances platelet aggregation by proteolytically activating receptors on platelets [76,77] In contrast, NETs counteract the effects of activated protein C (APC), which is a molecule known to inactivate the coagulation factors Va and VIIIa but can also destroy extracellular histones of NETs [78,79]. Pretreatment of neutrophils with APC before induction of NETosis reduces platelet adhesion to NETs [80]. Regarding the in-vivo relevance of NET effects on hemostasis, NE deficiency in mice or the application of NE inhibitors did not have a significant effect on NETosis, thrombus size, or frequency of deep vein thrombosis, which might be due to the redundancy of signaling pathways and effectors [81].

Considering the role of immune cells in the pathogenesis of abdominal aortic aneurysm (AAA), it has been shown that depletion of circulating neutrophils in mice inhibits experimental AAA development [82]. Furthermore, it has been reported that NETs are centrally involved in AAA pathogenesis, either in periodontal disease or in non-infectious causes of aneurysm formation: NET parameters such as citrullinated histones, cell-free DNA, or MPO-DNA complexes are elevated in the plasma and tissue of AAA patients [25,26,83]. NET constituents were found to be particularly prevalent in the adventitia as the outermost arterial vessel wall layer. Likewise, depositions of citrullinated histone 3 and histone 4 were shown in the intraluminal thrombus. Neutrophil extracellular traps are formed at early stages of experimental AAA in mice, as early as 2 to 3 days after aneurysm induction [25,83] Based on these findings, an anti-NET therapy was tested by applying DNase I, which was able to suppress aneurysm formation in experimental AAA mouse models [83,84] While DNase I attacks DNA as the backbone of NETs, the blockade of histone citrullination by chloro-amidines was tested as an alternative approach to NET reduction. Chloro-amidine suppresses NETosis by inhibiting the PAD enzyme family and it was shown that aneurysm formation was significantly reduced in this mouse model [25]. Interestingly, both DNase I and chloro-amidine provide additional protection against atherosclerosis by inhibiting NETosis in mice [21,67].

### 4.3. Autoimmunity and Autoinflammatory Diseases

Neutrophil extracellular traps are composed of intracellular components which are exposed to the immune system after expulsion [85]. Therefore, NETs are suspected to be involved in the initiation of autoimmune and autoinflammatory diseases. As common denominator, autoantibodies against NET components such as MPO-DNA complexes, citrullinated histones and neutrophil elastase are features of several systemic autoimmune diseases. This auto-directed immune response creates a vicious circle where NET components induce the production of autoantibodies, which subsequently bind to neutrophils, trigger NETosis, and aggravate the phenomenon [85]. Hence, neutrophil extracellular traps and their components could serve as potential future biomarkers for diagnosis and disease activity, or as therapeutic targets in autoinflammatory diseases [86]. NETs are proposed to play a key role in a variety of autoimmune diseases such as systemic lupus erythematosus, associated lupus nephritis, small-vessel vasculitis, psoriasis, rheumatoid arthritis, and diabetes mellitus type I [31,34,87,88]. Recently, chronic inflammatory bowel diseases such as Crohn’s disease and ulcerative colitis have also been associated with NET-driven inflammatory exacerbation, epithelial injury and increased thrombotic tendency [89]. For more details on this topic, Lee and Kronbichler and their colleagues provide a detailed review of NETs in autoimmune diseases [90].

Studies in patients and animal models with systemic lupus erythematosus (SLE) showed that oxidized mitochondrial DNA (mtDNA) is present in NETs. In combination with anti-mtDNA antibodies, this mtDNA, through the involvement of plasmacytoid dendritic cells, contributes essentially to the dysregulation of interferon α, which is critical in SLE patients [57,91]. In comparison to healthy study participants, a significant increase of mtDNA and anti-mtDNA antibodies in the serum of SLE patients was detected as well as evidence found that NETs and anti-mtDNA antibodies are associated with a higher incidence of lupus nephritis. In the SLE animal model, a therapeutic anti-NET approach was able to reduce the severity of the disease [57].

The antidiabetic drug metformin was recently proposed as a promising therapeutic option for NET-associated diseases, whose pleiotropic mechanisms of action include anti-inflammatory effects [92]. In-vitro experiments show a dose-dependent inhibition of NETosis when neutrophils are pretreated with metformin. To investigate the clinical significance of NETs and metformin in SLE, a randomized proof-of-concept trial with 113 SLE patients was performed. In patients with mild or moderate SLE, additional treatment with metformin after 12 months reduced not only the clinically manifest disease relapses by 51%, but also the required dose of corticosteroids [91]. These results suggest that anti-NET strategies may be a novel treatment approach for SLE.

Psoriasis as a chronic inflammatory dermatosis is characterized by the hyperproliferation of keratinocytes and the characteristic silvery-white plaques resulting from increased cell turnover. Neutrophils isolated from patient blood showed a higher tendency towards ROS-dependent NETosis compared to isolated neutrophils from healthy subjects [93]. Further studies demonstrated that neutrophil recruitment and NETs play an important role in psoriatic skin lesions. The pathogenetically and therapeutically central IL-17, released by neutrophils, can increase the neutrophil expression of defensins and of LL37, an antimicrobial peptide from the cathelicidin group. These molecules were shown to promote NETosis and inflammation in psoriatic plaques and other dermatopathological conditions [94,95,96,97].

Articular diseases such as rheumatoid arthritis or arthritis urica cause long-term cartilage and joint damage through persistent synovial inflammation or through the deposition of urate crystals. The resulting stimulated immune response and recruitment of neutrophils as well as the induced NETosis mostly favor the disease process [34]. In-vitro studies have shown that circulating and synovial neutrophils in patients with rheumatoid arthritis have a higher tendency to form NETs compared to healthy controls [30,94]. This could indicate pre-activation of neutrophils in chronic inflammatory conditions. Interestingly, alleviated symptoms have been associated with NETs in gout patients, as NETs contribute to the increased degradation of pro-inflammatory triggers due to the local accumulation of proteases on the expelled DNA network [95].

Regarding autoimmune reactions, NETosis can be considered as a source of autoantigens and may induce the formation of autoantibodies. In the case of rheumatoid arthritis, anti-citrullinated protein antibodies (ACPAs) are formed, which correlate with the activity of NETosis and neutrophil count [30]. Alternatively, NET formation can also be stimulated by neutrophil binding of so-called anti-neutrophil cytoplasmic antibodies (ANCAs) and anti-ribonucleoprotein (RNP) antibodies [31,96]. In the context of small-vessel vasculitis, which is characterized by a systemic vasculitis with associated necrosis and potential organ damage, it is known that antibodies to proteinase 3 and MPO can be detected frequently [97,98]. These cytoplasmatic enzymes of neutrophil granules are released during cell activation and NETosis and induce the formation of PR3-ANCA and MPO-ANCA, which subsequently contribute to vascular endothelial damage by activating the complement system. A feedback loop in the sense of a vicious circle is created by the fact that PR3-ANCA and MPO-ANCA in turn induce NETosis [34,99,100].

Diabetes mellitus type I is an autoimmune disease characterized by the progressive destruction of β pancreatic cells and leads to chronic hyperglycemia [101]. Decomposing β pancreatic cells present autoantigens that are recognized by autoreactive T cells, which subsequently leads to the production of specific (diagnostic) autoantibodies against glutamate decarboxylase, insulinoma-associated antigen 2 and zinc transporter 8 [102,103]. Patients with diabetes mellitus type I are at risk of developing neutropenia, either related to neutrophil sequestration in pancreatic tissue or through neutrophils infiltrating the islets of Langerhans, where NET formation is then stimulated by TNF-α. NETosis leads to further cytokine secretion and recruitment of neutrophils that enhance autoimmune events [34,104,105]. In the mouse model, NET formation in the islets of Langerhans was observed as early as the second postnatal week, while clinical studies in patients with diabetes mellitus type I showed an increased NETosis rate and a positive correlation to circulating NE. This may indicate a key role of neutrophils and NETosis in initiating autoimmunity in the pancreas [106].

An excess of proinflammatory mediators in the multifactorial and complex pathogenesis of the chronic inflammatory bowel diseases, Crohn’s disease and ulcerative colitis, leads to an overshooting immune reaction and as a result the bowel wall undergoes pathological changes which characteristically manifest themselves as intermittent acute flare-ups and symptom-free phases [107,108,109]. It was shown that the expression of NET-associated proteins such as PAD4 is increased in colon biopsies of patients with Crohn’s disease and ulcerative colitis compared to healthy control subjects. Further data suggest that TNF-α promotes NET release and that therapy with Infliximab, a high-affinity monoclonal antibody to TNF-α, may reduce PAD4 expression and NET formation [35]. A recent study based on a murine model demonstrated both, a reduction in NET release as well as colitis and decreasing colitis-associated tumorigenesis when treating with DNase I. Furthermore, a reduced thrombus formation and platelet activation was observed [89].

### 4.4. Metabolic Diseases

The increasing global incidence of obesity and metabolic diseases such as diabetes mellitus type II favors inflammatory conditions in which the innate immune response is activated and NETosis contributes to further deregulation of the immune system, oxidative stress, inflammation, complications of metabolic disorders, and hyperglycemia [34,110,111,112]. Studies have shown that morbid obesity is associated with chronic inflammation and increased neutrophil activity as well as increased NET formation and ROS production. Plasmatic NET parameters such as MPO-DNA complexes were higher in obese patients than in the control group and correlated with body weight, body mass index, waist and hip circumference, glucometabolic parameters, and systolic blood pressure [112,113,114]. Interestingly, also in animal models neutrophils from mice fed a high-fat diet were more prone to spontaneous NETosis than those from mice on a low-fat diet [115].

Diabetes mellitus type II as a metabolic disease is mainly due to lifestyle factors and genetics and is characterized by the development of hyperglycemia and cellular insulin resistance [116]. In this context, increased NETosis was found in diabetes type II patients compared to healthy controls. When these patients were treated with the antidiabetic agent metformin, a significant reduction or normalization of hyperglycemia was observed six months after the start of therapy, while NET parameters, IL-6 and TNF-α blood concentrations reached normal values only after 12 months. This is an indication that NETosis in type II diabetes patients is not necessarily the result of impaired blood glucose control, but is primarily associated with pro-inflammatory cytokines [117]. The main triggers for diabetic vascular complications are hyperglycemia, increased production of mitochondrial ROS and lipotoxicity, which are also partly caused by NET release and the connected activation of NADPH oxidase and mitochondrial ROS [104,118,119]. One study demonstrated a positive correlation between the occurrence of diabetic organ damage, such as nephropathy or atherosclerotic cardiovascular disease, and cell-free DNA as a NET parameter. In addition, it was shown that plasmatic histone concentrations correlated directly with the HbA1c value [32]. Further research efforts are needed to sketch a more detailed picture of the regulation and role of NETs in diabetes mellitus type II, as discrepant in-vitro results showed that hyperglycemia can also possibly impair and delay NET formation [120].

### 4.5. Malignant Neoplasia

Neutrophils play a multifaceted role in tumor biology and there is growing evidence that NETs are also fundamentally active in the pathogenesis of tumors [121]. The granulocyte colony-stimulating factor (G-CSF) is upregulated in many tumor entities, leading to an increase in systemic neutrophil activation and NET formation, as well as an increase in NET-associated complications such as thrombosis. However, tumor-associated thrombosis is connected with a poor prognosis and represents the second most frequent cause of death in cancer patients after metastasis [122,123]. As a mechanistic link between malignant tumors and thrombosis, for example, there is the activation of the alternative complement pathway and the release of lipopolysaccharide by cell membrane damage in intestinal tumors. Circulating lipopolysaccharide and activated complement factors stimulate NETosis (Figure 1), which promotes blood coagulation [9,124]. In the context of neoplasia, NETs have also been proposed as mediators of metastasis by “trapping” circulating tumor cells and facilitating their accumulation in peripheral tissues and by “reawakening” of dormant cancer cells through NET-associated proteins [125,126]. In addition, neutrophils can promote metastasis via NET-independent mechanisms, such as interaction with leukotrienes, which propagate metastasis-initiating cells with high tumorigenic potential and therefore support the colonization of malignant cells in foreign tissues [127,128]. Therapeutic approaches to block NETs are of considerable research interest in the tumor field and have shown promising results. For example, NET formation stimulated the invasion and migration of breast cancer cells in-vitro, while NET inhibition by DNase I blocked these processes and significantly reduced lung metastases in a corresponding mouse model [37]. Likewise, therapeutic effects of DNase I on metastasis in pancreatic carcinomas were shown in-vivo using an orthotopic xenograft mouse model [129]. In patients with colorectal or breast cancer, NETs were found more frequent in patients with liver metastases, and in the case of early breast cancer stages, the increased incidence of NETs in patient serum was found a suitable biomarker for the prediction of liver metastases [130]. The implications of NETs in cancer and cancer-associated pathologies have been extensively reviewed by Masucci et al., Garley et al. and Cedervall and Olsson [131,132,133]

### 4.6. Sepsis

The estimated annual incidence of sepsis is 18 million patient cases worldwide with a mortality rate between 30% and 50% [134,135]. NET formation has both, beneficial and detrimental aspects, although it remains unclear which aspect predominates [136]. On the one hand, NETs act as a valuable antimicrobial defense mechanism and on the other hand, NETs lead to organ failure and even death when regulatory mechanisms fail. In the early phase of sepsis, NETs contribute positively by capturing and eliminating pathogens. However, subsequently the systemic infection leads to tissue and endothelial damage, which can ultimately result in wide-spread thrombosis and disseminated intravascular coagulation [136]. Clinical parameters such as the *Sequential Organ Failure Assessment* (SOFA) score for organ dysfunction and the *Acute Kidney Injury Network* (AKIN) definition of acute kidney damage correlate positively with the NET parameter of cell-free DNA, measured in septic patient serum [137]. In addition, in the murine model it has been shown that binding of a monoclonal antibody to NET complexes in sepsis increases the overall survival. This seems mainly due to the fact that antibody binding protects NETs from DNase I, resulting in less toxic NET-associated degradation products [138].

### 4.7. COVID-19

Currently, an association between NETs and the severe acute respiratory syndrome coronavirus 2 (SARS-CoV-2), which causes the coronavirus disease 2019 (COVID-19), is being discussed [139]. COVID-19 manifests itself with flu-like symptoms and a viral pneumonia and can lead to acute lung failure, more precisely to an acute respiratory distress syndrome (ARDS), and multiple organ failure [140]. Elevated levels of NET parameters, such as cell-free DNA, MPO-DNA complexes, and citrullinated histone H3, were detected in the blood serum of COVID-19 patients. Interestingly, cell-free DNA and MPO-DNA complexes were also found at significantly higher levels in hospitalized, mechanically ventilated patients compared to hospitalized, non-ventilated patients [141]. Clinical cases of severe COVID-19 disease suggest that initial vascular damage and subsequent organ dysfunction are associated with excessive NET formation. Histopathologically, microangiopathic occlusions by aggregated NETs are detected in COVID-19 patients. The smallest pulmonary vessels, especially along the alveolar septa are mainly affected. Further analyses revealed the pathological presence of aggregated NETs and their main components also in blood vessels of the kidney and liver, in particular in the glomeruli and the hepatic periportal fields [142]. The overly activation of neutrophils and the associated excessive NET formation in COVID-19 patients highlight NETs as a possible therapeutic target for the immunopathological complications of critically ill COVID-19 patients. The development of novel anti-NET therapeutic strategies might help to reduce morbidity and mortality of COVID-19 [143,144].

## 5. Current Perspective and Future Direction

Neutrophils, as an essential component of the innate immune system, play a crucial role in the control of infectious diseases. In addition, they are involved in the pathogenesis of various diseases that have an inflammatory component. Current research suggests that NETs may be central mediators of these processes. NET formation may foster chronic inflammation, thereby promoting cardiovascular and autoimmune diseases as well as increasing the risk or progression of cancer. Therefore, NETs represent an interesting target for therapeutics in the management of different disease entities. Although therapeutic application of DNase I is already clinically relevant in various other diseases, the utility in disrupting rather than preventing NETs by DNase I remains controversial. Other therapeutic approaches that block the release of NETs such as inhibition of PAD4, histone neutralization, ROS scavenging or partial clearance of neutrophils are currently limited to the preclinical stage. Due to the potentially low immunosuppression by anti-NET therapy, the success of a cost-effective treatment would be particularly advantageous. Indeed, it will be necessary to further investigate and understand the regulation and balance of NET induction, inhibition, and degradation to pharmacologically target NETs, without compromising the patient’s immune defense. In order to enter clinical practice, anti-NET therapy requires further research efforts to understand the detailed functions and effects of NETs on health and disease in view of the heterogeneous disease patterns.

## Figures and Tables

**Table 1:** Triggers of NET formation in disease.

Category	Disease	Identified Triggers
Cardiovascular	Atherosclerosis	IL-8 [20], IL-1β, crystalized cholesterol [21], oxLDL [22]
	Thrombosis	HMGB-1 [23], pathophysiologic hemodynamic forces [24]
	Abdominal aortic aneurysm	IL-1β [25], *Porphyromonas gingivalis* [26]
Autoimmune & Autoinflammatory	Systemic lupus erythematosus & lupus nephritis	IFN-α, ANCAs, RNPs [27], acetylated histones & apoptotic microparticles [28]
	Psoriasis	IL-8, IL-17, IL-23, TNF-α, autoantigens (keratin 17, LL37) [29]
	Rheumatoid arthritis & arthritis urica	ACPAs, ANCAs, RNPs, IL-17A, TNF-α [30], hyperuricemia and monosodium urate crystals [19]
	Small-vessel vasculitis	ANCAs [31]
	Diabetes mellitus type I	Hyperglycemia [32], β-cell death & autoantibodies [33]
	Crohn’s disease & ulcerative colitis	enhanced ROS production [34,35]
Metabolic	Obesity & diabetes mellitus type II	Hyperglycemia [32], IL-6, IL-8, TNF-α, homocysteine [36]
Malignant neoplasia	metastatic breast cancer cells, metastatic colorectal cancer	G-CSF [37], IL-8 [38], intratumoral hypoxia [39]
Infectious	Sepsis	HMGB-1 [40], eCIRP [41], oxLDL [42]
	COVID-19	HMGB-1 [43], hypoxia [44]

IL: interleukin; HMGB-1: high-mobility group box 1 protein; IFN-α: interferon α; ANCA: anti-neutrophil cytoplasmic antibodies; RNP: anti-ribonucleoprotein (antibodies); TNF-α: tumor necrosis factor-α; ACPA: anti-citrullinated protein antibodies; ROS: reactive oxygen species; G-CSF: granulocyte colony-stimulating factor; eCIRP: extracellular cold-inducible RNA-binding protein; oxLDL: oxidized low-density lipoprotein.

## Data Availability

No new data were created or analyzed in this review. Data sharing is not applicable to this article.

## Abbreviations

AAA	abdominal aortic aneurysm
ACPAs	anti-citrullinated protein antibodies
AKIN	Acute Kidney Injury Network
ANCA	anti-neutrophil cytoplasmic antibody
APC	activated protein C
ApoE	apolipoprotein E
ARDS	acute respiratory distress syndrome
eCIRP	extracellular cold-inducible RNA-binding protein
G-CSF	granulocyte colony-stimulating factor
HMGB-1	high-mobility group box 1 protein
IFN-α	interferon α
IL	interleukin
MPO	myeloperoxidase
mtDNA	mitochondrial DNA
NE	neutrophil elastase
NET	neutrophil extracellular trap
Nox2	NADPH-oxidase 2
oxLDL	oxidized low-density lipoprotein
PAD4	protein arginine deiminase 4
PMA	phorbol-12-myristate-13-acetate
RNP	anti-ribonucleoprotein
ROS	reactive oxygen species
SARS-CoV-2	severe acute respiratory syndrome coronavirus 2
SLE	systemic lupus erythematosus
SOFA	Sequential Organ Failure Assessment
TNF-α	tumor necrosis factor-α
